# Global Isomeric Survey of Elusive Cyclopropanetrione: Unknown but Viable Isomers

**DOI:** 10.3389/fchem.2019.00193

**Published:** 2019-04-03

**Authors:** Jing-fan Xin, Xiao-ru Han, Fei-fei He, Yi-hong Ding

**Affiliations:** ^1^Laboratory of Theoretical and Computational Chemistry, Institute of Theoretical Chemistry, Jilin University, Changchun, China; ^2^Inner Mongolia Key Laboratory of Photoelectric Functional Materials, College of Chemistry and Chemical Engineering, Chifeng University, Chifeng, China

**Keywords:** neutral cyclopropanetrione, C_3_O_3_, global isomeric survey, kinetic stability, computational study, high-energy density materials, alkali metal-ion battery

## Abstract

Despite the great interest in energy storage application, stable neutral C_n_O_n_ (*n* > 1) structures either in thermodynamics or kinetics have yet been largely limited due to the rather high tendency to release the very stable CO molecule. The neutral cyclopropanetrione (C_3_O_3_) cluster has long remained elusive since no isomer with sufficient kinetic stability has been found either experimentally or theoretically. In this work, we constructed the first global potential energy surface of singlet C_3_O_3_ at the CCSD(T)/aug-cc-pVTZ//B3LYP/aug-cc-pVTZ level, from which the kinetic stability of a wide range of C_3_O_3_ isomers can be determined by investigating their isomerization and fragmentation pathways. Amongst, a three-membered ring structure **01** is the global C_3_O_3_ isomer with a barrier of 10.6 kcal/mol at the sophisticated W1BD level. In particular, two carbene-type isomers **02** and **04** possess appreciable destruction barriers of 20.3 and 24.7 kcal/mol at W1BD, respectively. Thus, **02** and **04** can be useful building blocks for constructing larger high-energy density carbon-oxygen clusters. Moreover, with the carbene center, both might effectively functionalize various nano-materials while retaining the electrochemical active carbonyl and epoxyl moieties that are very desirable in alkali metal-ion batteries.

## Introduction

Carbon (C) and oxygen (O) are key elements on earth and in space. Clusters constituted simply by them form a special class of oxides of carbon, namely oxocarbons(Rubin and Gleiter, 2000; Horiuchi et al., 2010; Kikuchi et al., 2014; Davis and Sajeev, 2017; Wang et al., 2018a). The huge energy release from C_n_O_n_ to nCO provides great promise that a kinetically stabilized C_n_O_n_ might find applications in the so-called high-energy density materials (HEDMs) (Schmidt et al., 2000; Gambi et al., 2001; Corkran and Ball, 2004; Xia et al., 2017). In fact, the polymeric CO networks as potential HEDMs have been computationally predicted to exist under the high-pressure environments (Lipp et al., 2005; Ryu et al., 2016, 2017). Yet finite-sized C_n_O_n_ clusters with both high-energy and appreciable kinetic stability against destruction (i.e., isomerization/fragmentation) still remain unknown. Moreover, there have been growing evidences that the rich oxygen density in form of carbonyl and epoxyl groups are key in development of alkali metal-ion electrodes for sustainable ion batteries (Chen et al., 2009; Seo et al., 2011; Kim et al., 2014; Zhao et al., 2016; Larm et al., 2018; Wang et al., 2018b). Kinetically stable C_n_O_n_ that natively bear rich oxygens would surely find interest in such applications.

The structure, bonding and stability of chemically bound C_n_O_n_ have been explored in a large number of publications (Frenking, 1990; Schröder et al., 1998, 1999; Talbi and Chandler, 2000; Sabzyan and Noorbala, 2003; Zhou et al., 2010; Bao et al., 2012; Guo et al., 2012; Hu et al., 2012; Dixon et al., 2015; Liu et al., 2015; Hansen et al., 2016). To our surprise, larger CO oligomers in poly-cyclic form with *n* = 8, 9, 10, 12 were reported early (in 1967 and 1984; Verter and Dominic, 1967; Nallaiah, 1984). Their easy accessibility is in accordance with their good thermodynamic stability with respect to nCO (Schröder et al., 1999), and these low-lying clusters surely cannot be used for HEDMs. However, the detection and characterization of C_n_O_n_ with intermediate size has been found quite difficult, partly due to their worse thermodynamic stability (Schleyer et al., 2000). The possible existence of C_2_O_2_ was suggested more than 200 years ago (Staudinger and Anthes, 1913). Yet the combined spectroscopic and theoretical study has shown that the most feasible isomer, triplet linear OCCO, could only be transient or fleeting due to the low intersystem crossing barrier 3.0 kcal/mol (Schröder et al., 1998). Up to now, a conclusive spectroscopic characterization of OCCO still remains missing (Dixon et al., 2015; Lunny et al., 2018). For the monocyclic structures (*n* = 3–6), their generation and characterization were reported recently in the negative ion photoelectron (NIPE) mass spectroscopic studies (Hsu and Lin, 1978; Guo et al., 2012; Bao et al., 2013; Chen et al., 2014). We must note that the observed monocyclic C_3_O_3_ was just a hilltop structure with two imaginary frequencies (Chen et al., 2014). In spite of the available structural and thermodynamic information (Sabzyan and Noorbala, 2003; Sahu and Lee, 2005; Zhou et al., 2010; Bao et al., 2012; Liu et al., 2015; Hansen et al., 2016), to our best knowledge, very little study has been made to address the kinetic stability of C_n_O_n_ (*n* > 2) isomers, i.e., their lowest barriers against isomerization/fragmentation.

**Scheme 1:**
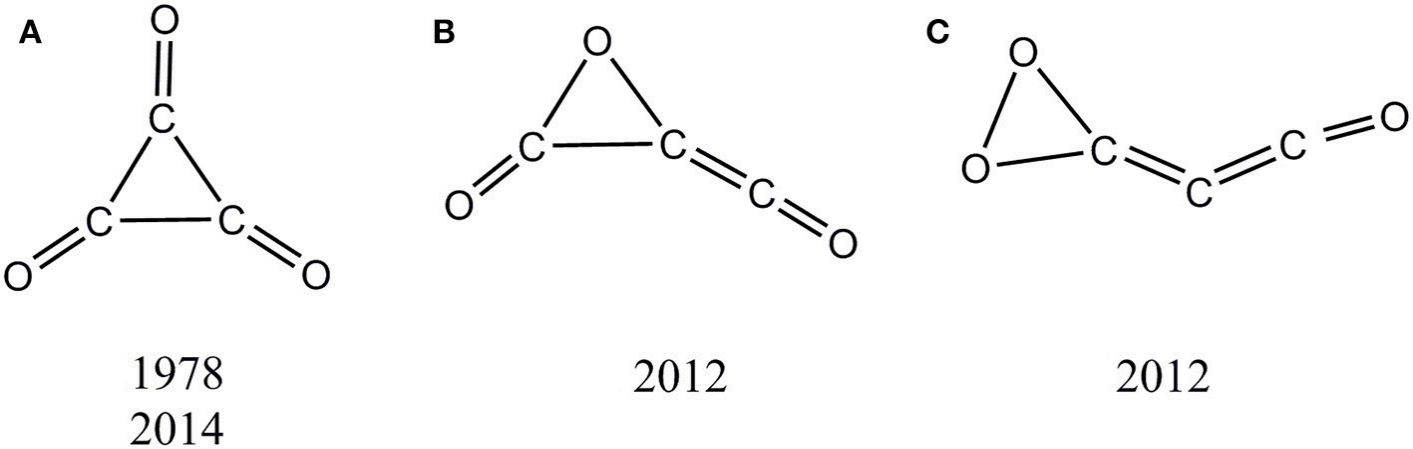
Proposed singlet C_3_O_3_ isomers in literatures with its first proposed year and characterized year. **(A)** C_3_-ring isomer. **(B)** C_2_O- ring isomer. **(C)** CO_2_- ring isomer.

In this work, we focus on the cyclopropanetrione cluster (C_3_O_3_) that could present the smallest mono C_3_-ring. Three distinct types of singlet C_3_O_3_ isomers (**A**–**C** in Scheme 1) have been reported in literatures (Hsu and Lin, 1978; Hu et al., 2012; Chen et al., 2014). The long expected and hotly studied monocyclic singlet isomer **A** is actually a second-order saddle point, and its observation can only be realized under very extreme experimental conditions (e.g., NIPE; Chen et al., 2014). The isomers **B** and **C** of C_3_O_3_ were computationally reported in 2012 (Hu et al., 2012), yet their kinetic stability is still uncertain. We can say that at present, no C_3_O_3_ with reasonable kinetic stability has been shown either experimentally or computationally. Thus, C_3_O_3_ represents a quite “elusive” oxocarbon system.

Surely, the key to resolve this problem is to build a global potential energy surface (PES) picture of C_3_O_3_, which involves both the C_3_O_3_ isomers and the isomerization/fragmentation transition states as many possible. With the PES, the kinetic stability of each C_3_O_3_ isomer can then be determined. Unfortunately, building the global PES for such a high-energy and hexatomic molecule should be time-consuming, tedious and even exhaustive. In the present work, applying an effective global PES search strategy, we computationally constructed the first global potential energy surface (PES) of singlet C_3_O_3_, which helped us determine the kinetic stability of a wide range of isomers. Amongst them we for the first time identified the global isomer of C_3_O_3_, i.e., **01**. In particular, two carbene-like isomers **02** and **04** have the potential use in energy storage applications (e.g., HEDM and ion batteries). Such a thorough PES would provide a good base for future theoretical and laboratory studies of these C_3_O_3_ isomers.

## Computational Details

The search of isomers and transition states of singlet C_3_O_3_ was carried out on a locally developed platform “global potential energy surface survey (GPESS)” (Shao and Ding, 2010; Ding, 2015; Bo and Ding, 2018). The flow chart of our strategies for constructing C_3_O_3_ PES is shown in the Supporting Information (SI_1). The isomeric search was based on the grid search program “grid-based isomeric search strategy” at the B3LYP/6-31G(d) level (Parr and Yang, 1989; Becke, 1992; Perdew et al., 1992) for both geometries and frequencies. The transition state (TS) search was divided into two types, i.e., the isomeric conversion and the isomeric decomposition. For the interconversion TS search, the “QST2” algorithm (Foresman and Frisch, 1996; Hrarchian and Schlegel, 2005), was applied, which yet has a difficulty in placing atoms in the same atomic order between reactant and product (especially for molecules with many homo-atomic elements). This was treated in GPESS by automatic enumeration of all possible combinations. Besides, the decomposition TS search was considered directing to some relatively stable molecular fragments like CO and CO_2_. The connection of each transition state was determined by the intrinsic reaction coordinate (IRC) (Fukui, 1981) calculations. The effectiveness of such isomeric and TS search strategies has been confirmed in study of various small to medium-sized systems (Cui et al., 2011; Gao and Ding, 2012; Tang et al., 2012; Guo et al., 2013a,b, 2014; Zhang and Ding, 2014, 2015; He and Ding, 2016; Xu et al., 2017; Bo et al., 2019).

Further, the geometry and frequencies of each isomeric and transition state structure were refined at the B3LYP/aug-cc-pVTZ optimization level followed by the CCSD(T)/aug-cc-pVTZ single-point energy calculations. The eventual energy includes the Gibbs free energy corrections (GFEC). The overall were included in CCSD(T)/aug-cc-pVTZ single-point energy calculation. For all the obtained isomers, we carried out the “stability” analysis of the wave-function at the B3LYP/aug-cc-pVTZ level, applying the broken-symmetry strategy of Noodleman (Noodleman, 1981; Noodleman and Davidson, 1986). For key species, the composite CBS-QB3 (Montgomery et al., 1999, 2000) and W1BD (Barnes et al., 2009) methods were applied to get more reliable energy. All the calculations were carried out with the GAUSSIAN03 and GAUSSIAN09 program packages (Frisch et al., 2004, 2013).

## Results and Discussions

### Potential Energy Surface of Singlet C_3_O_3_

**Figure 1:**
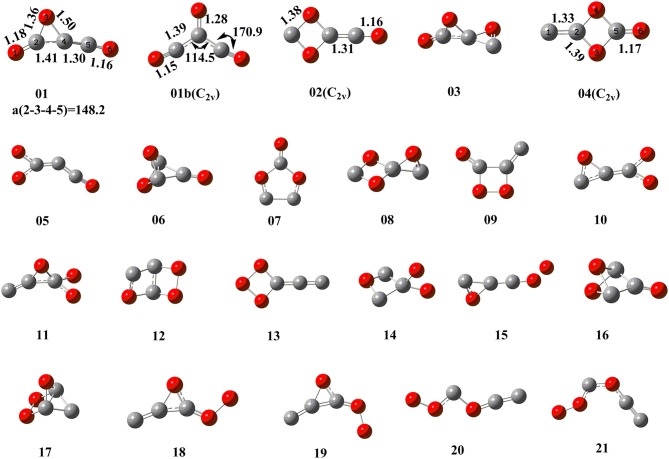
Structural information of singlet C_3_O_3_ isomers at the B3LYP/aug-cc-pVTZ level.

**Figure 2:**
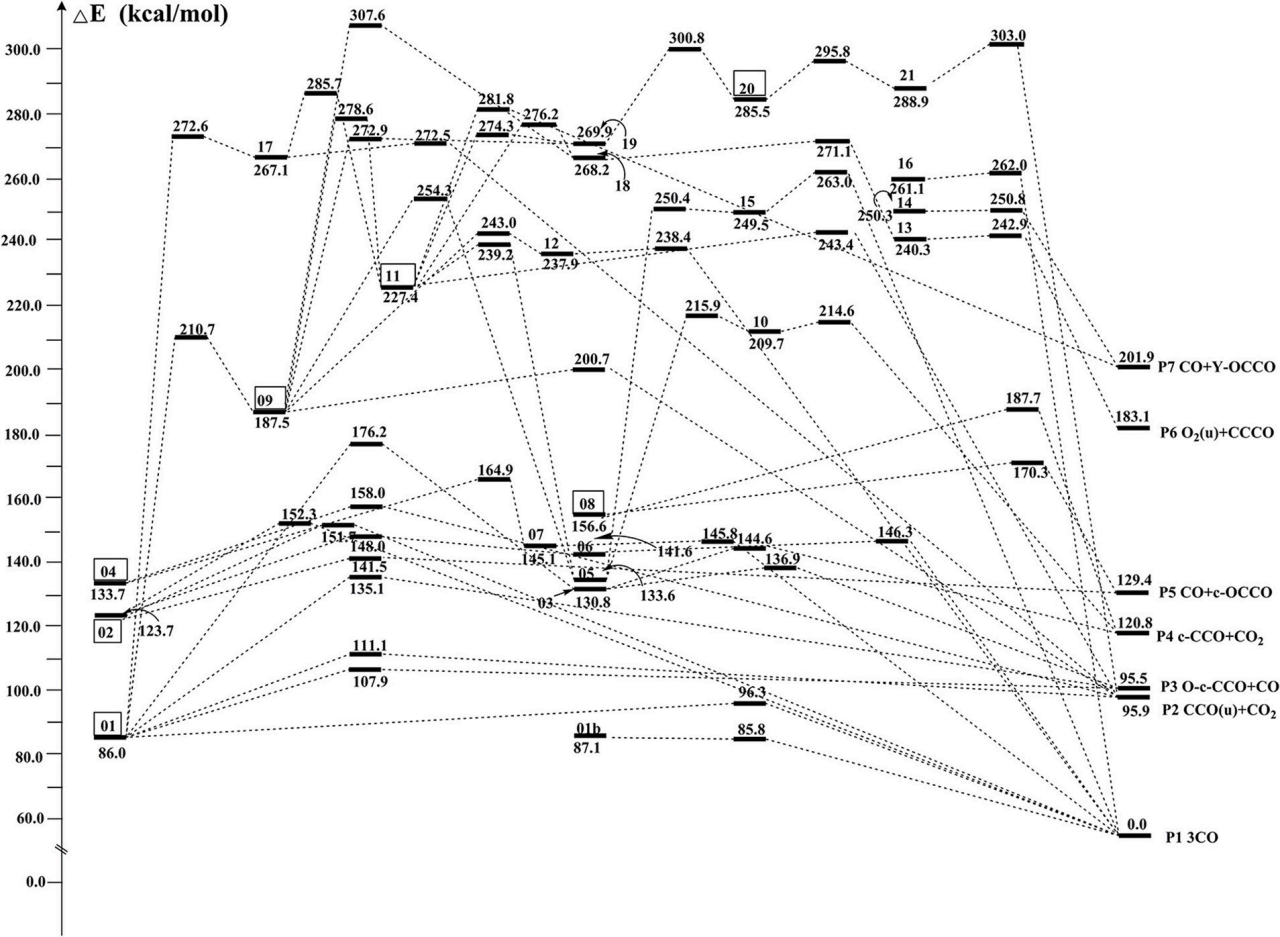
Schematic potential energy surface of singlet C_3_O_3_ at the CCSD(T)//B3LYP+GFEC level.

By means of the extensive “grid” isomeric search and the transition state search strategies, we eventually located a total of 22 singlet chemically bound isomers and 46 transition states (Supporting Information, SI_2, SI_3). Note that our study found many other transition states whose imaginary frequency is only associated with the evolution of one separate part, while the other part is just a spectator. These transition states are not related to the evaluation of the kinetic stability of C_3_O_3_ isomers and thus not discussed. The relative energies (RE) with respect to **P1** 3CO (0.0) and the corresponding destruction barriers of each singlet isomer are listed in Table 1. For simplicity, “CCSD(T)//B3LYP+GFEC” represents the CCSD(T)/aug-cc-pVTZ//B3LYP/aug-cc-pVTZ values with GFEC. The structures of singlet isomers are listed in Figure 1. The schematic potential energy surface of singlet C_3_O_3_ isomers is given in Figure 2. Energies for singlet C_3_O_3_ isomers, transition states and products are shown in **SI**_**4**, **SI**_**5**, **SI**_**6**. The natural molecular orbitals for **01**, **02**, **04** are shown in **SI**_**8**.

**Table 1:** RE values for singlet C_3_O_3_ isomers with respect to 3CO as well as the destruction barriers for each isomer at the CCSD(T)//B3LYP+GFEC level.

**Isomer**	**RE (kcal/mol)**	**Destruction barrier (kcal/mol)**	**Isomerization/decomposition product**
**01**	86.0 (83.0^a^, 84.1^b^)	10.3 (10.5^a^, 10.6^b^)	**P1** 3CO
**01b**	87.1	−1.3	**P1** 3CO
**02**	123.7 (121.8^a^, 122.9^b^)	17.8 (21.5^a^, 20.3^b^)	**P5** c-OCCO+CO
**03**	130.8	6.1	**P3** O-c-CCO+CO
**04**	133.7 (132.6^a^, 133.4^b^)	24.3 (24.5^a^, 24.7^b^)	**P2** (u) CCO+CO_2_
**05**	133.6	1.5	**01**
**06**	141.6	4.7	**P1** 3CO
**07**	145.1	0.7	**P1** 3CO
**08**	156.6	13.7 (14.4^a^)	**P5** c-OCCO+CO
**09**	187.5	13.2 (12.8^a^)	**P2** (u) CCO+CO_2_
**10**	209.7	4.9	**P4** c-CCO+CO_2_
**11**	227.0	12.2 (12.0^a^)	**05**
**12**	237.9	0.5	**P1** 3CO
**13**	240.3	2.6	**P6** (u) O_2_+CCCO
**14**	250.3	0.5	**P7** Y-OCCO+CO
**15**	249.5	0.9	**05**
**16**	261.1	0.9	**P1** 3CO
**17**	267.1	5.4	**P2** (u) CCO+CO_2_
**18**	268.2	2.9	**13**
**19**	269.9	3.0	**09**
**20**	285.5	10.3	**21**
**21**	288.9	6.9	**20**

a *The Gibbs free energy values at CBS-QB3*.

b *The Gibbs free energy values at W1BD*.

### Global Isomer

**Scheme 2:**
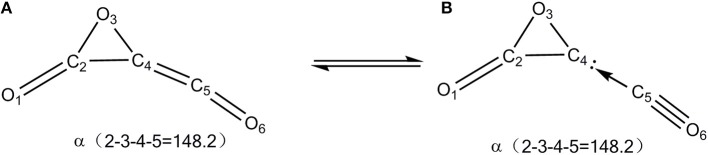
Two resonant structures of **01**. **(A)** Cumulenic type. **(B)** Donor-acceptor type.

The global C_3_O_3_ isomer is **01**, which lies 86.0 kcal/mol higher than the fragments 3CO (0.0). This indicates that C_3_O_3_ is truly an energized system. As shown in Figure 1, the exocyclic C_(4)_-C_(5)_ bond distance of **01** is 1.30 Å, indicative of the ketene-like >C = C = O bonding. To our surprise, C_(4)_ is somewhat pyramidal in contrast to the usual sp^2^-C, for which three connected bonds are in a plane. So, **01** should have contribution from two resonant structures (A) >C = C = O and (B) >C(:)← CO (see Scheme 2).

To test the bonding picture **B** in isomer **01**, we computed the three-membered ring (3MR) C_2_O_2_ when the exocyclic CO is formally removed from **01**. The 3MR C_2_O_2_ is truly a singlet local minimum structure and is electronically stable during the “stability” analysis. Its triplet structure does not exist, which upon optimization would collapse to the ground structure, i.e., triplet OCCO. The electronic stability of the unsaturated carbon in 3MR C_2_O_2_ must originate from the lone-pair electronic stabilization of the neighboring O-atom. So, C_(4)_ in **01** has the significant closed-shell divalent carbene character with one electron lone pair (:) and one vacant orbital that is occupied by the Lewis base CO. This bonding picture is supported by two facts. First, the leaving barrier of CO (22.5 kcal/mol) is roughly quarter of the typical C-C single bond energy (85.4 kcal/mol; Sanderson, 1983) and the reverse association barrier between CO and 3MR-C is negligibly tiny. Note that the barrier becomes negative as −1.3 kcal/mol at the CCSD(T)//B3LYP+GFEC level, which is just a result of the single-point energy calculation at a lower-level geometry. This phenomenon usually occurs for low-barrier processes. This is well-indicative of the donor-acceptor interaction as shown in B). Second, there is an appreciable binding energy between the 3MR-C atom and the Lewis acid AlCl_3_ (12.6 kcal/mol at the B3LYP/aug-cc-pVTZ+ZPVE level), suggestive of the existence of an electron lone pair on C_(4)_.

### Viable and Fleeting Isomers

According to Hoffmann et al. (2008), a viable molecule should be resistant to fragmentation, isomerization, and dimerization or higher chemical aggregation. For a gas-phase molecule like C_3_O_3_, the aggregation can usually be omitted (the chance of bimolecular association is very little). So, the kinetic stability against both fragmentation and isomerization is the key to determine the lifetime of a C_3_O_3_ structure. Species with several kcal/mol should better be viewed as fleeting or transient. For safety, in this work, the value 10 kcal/mol is artificially taken as the bar of “fleeting” or “transient.”

Among the 22 located singlet C_3_O_3_ isomers, a total of seven isomers (**01**, **02**, **04**, **08**, **09**, **11**, **20**) were identified to have the destruction barriers of ≥10 kcal/mol. The easiest conversion pathway for the global isomer **01** is to decompose into **P1** 3CO with a marginal barrier of 10.3 kcal/mol. Two four-membered ring (4MR) isomers **02** and **04** (lie at 123.7 and 133.7 kcal/mol, respectively) both contain a C(μ-O)_2_C = C core (μ for “bridge”). In particular, both **02** and **04** feature the unsaturated carbenes. Their most feasible primary pathways are different, i.e., **P5** CO + c-OCCO for **02** and **P2** (u)CCO + CO_2_ for **04** with the corresponding barriers of 17.8 and 24.3 kcal/mol. Isomers **08** and **11** (at 156.6 and 227.0 kcal/mol, respectively) are bicyclic and spiral. Their most feasible product is **P5** CO+c-OCCO via the direct and indirect pathways with the corresponding barriers 13.7 and 12.2 kcal/mol. For isomer **09** with bridge-OO, the most favorable product is **P2** (u)CCO + CO_2_ with the barriers 13.2 kcal/mol. The very high-energy ***cis***/***trans*** chainlike isomers **20** (285.5 kcal/mol) and **21** (288.9 kcal/mol) both have a terminal-OO and can be interconverted to each other. As shown in SI_9, **21** might undergo the intersystem crossing during fragmentation to CCO (triplet)+COO, which greatly decrease its kinetic stability (<10 kcal/mol). By contrast, the interconversion governs the kinetic stability of **20** and its the destruction barrier is 10.3 kcal/mol.

The remaining 15 singlet C_3_O_3_ isomers have smaller destructions barriers of ≤10 kcal/mol, i.e., **01b** (-1.3), **03** (6.1), **05** (1.5), **06** (4.7), **07** (0.7), **10** (4.9), **12** (0.5), **13** (2.6), **14** (0.5), **15** (0.9), **16** (0.9), **17** (5.4), **18** (2.9), **19** (3.0), **20** (6.9). The values in () are the respective destruction barriers in kcal/mol. Amongst them isomer **20** has the largest destruction barrier 6.9 kcal/mol. Clearly, the 15 isomers should be considered as “fleeting” or “transient.” Note that isomer **01b** has a physically troublesome negative barrier height value −1.3 kcal/mol according to the transition state theory, although the barrier height is a reasonable one (0.2 kcal/mol) at B3LYP/aug-cc-pVTZ+GFEC level. This is an indication that **01b** is either not a minimum or faces a negligibly small barrier at the CCSD(T) level.

To determine whether a single-reference-based electron correlation procedure (here is CCSD(T)/aug-cc-pVTZ) is appropriate or not, the T1 diagnostic values (T1Diag) were computed. A large T1 (i.e., >0.02) probably indicates that a multireference electron correlation procedure is needed (Lee and Taylor, 1989). In our work, the T1Diag values lie below the threshold 0.02 for key isomers and transition states (see SI_7). Understandably, for the –OO isomers **20** and **21**, the T1Diag values are greater, i.e., around 0.04 for isomers and around 0.03 for transition states. Other species have acceptable T1Diag values of around 0.02 except **ts04/07**, **ts08/P1**, **uts09/P2**, and **ts11/P2**, whose T1Diag values are close to 0.03.

In the present study on the C_3_O_3_ PES construction as well as the composite CBS and W1BD calculations were all based on the B3LYP method. We thus further performed the comparative study of B3LYP with B3LYP-D3BJ (Grimme et al., 2011) and B2PLYP-D3(Grimme, 2006; Grimme et al., 2010) using the same aug-cc-pVTZ basis set followed by the CCSD(T)/aug-cc-pVTZ single-point energy calculations for both the isomeric and destruction transition state strucutures for key structures, i.e., **01**, **02**, **04**, **01-TS**, **02-TS**, and **04-TS** (see SI_10, SI_11). For the geometries (bond length, bond angle, dihedral angle), B3LYP agrees excellently with B3LYP-D3BJ and B2PLYP-D3 for **02**, **04**, **02-TS**, and **04-TS**. The deviation is relatively larger for **01** and **01-TS** with largest differences value of bond length, bond angle and dihedral angle are 0.11 Å, 4.2°, and 10.5°, respectively. This must be due to the unique electronic structure of **01** as bearing two resonant structures >C = C = O and >C(:)← CO (as discussed above). Yet the relative energies and destruction barriers at CCSD(T)//B3LYP agree quite well with those at CCSD(T)//B3LYP-D3BJ and CCSD(T)//B2PLYP-D3 within 1.0 kcal/mol for all the three isomers.

### Implications

Let us compare our extensive potential energy surface study with literatures. Only three singlet C_3_O_3_ structures (**A**, **B**, **C**) have been proposed (see Scheme 1). The second-order saddle point nature of **A** was reproduced in our work. The structures **B** and **C** predicted as local minima in 2012 at B3LYP/6-31G(d) level correspond to **01** and **05**, respectively in our work. To our great surprise, **01** (**B**) was reported to lie by 34.9 kcal/mol higher in energy than **05** (**C**) in the 2012 work, in sharp contrast to the present study that **01** (**B**) is the global minimum and 47.6 kcal/mol more stable than **05** (**C**). After careful repetition and analysis, the reason for such dramatic discrepancy was found to be that the 2012 study actually used the results at two different levels for comparison, i.e., B3LYP/6-31G (with no d function) for **01** (**B**) and B3LYP/6-31G(d) **05** (**C**). Thus, the present work for the first time predicted **01** as the global minimum of C_3_O_3_.

The two carbene-like isomers **02** and **04** with good kinetic stability deserve special attention. First, they provide much promise to design larger oxocarbon clusters with similar structural backbones. Second, the intrinsic carbene-reactivity allows them to be used in functionalizing various nano-materials such as graphenes (Bruce, 1991; Rit et al., 2016; Meyer, 2018). In particular, both isomers have one carbonyl (C = O) and two epoxyl (O<) groups, which could make them promising in the alkali metal-ion batteries (Wang et al., 2013; Zan, 2014; Deng et al., 2017; Zhao et al., 2017).

In particular, the destruction barrier 24.3 kcal/mol of the isomer **04** is quite close to those of the already synthesized species, e.g., N_2_CO (25.8 kcal/mol; Korkin et al., 1996) and pentazole anion ${\text{N}}_{5}^{-}$ (25.2 kcal/mol; Rahm and Brinck, 2010). With very huge energy release to 3CO (133.7 kcal/mol), **04** deserves to be taken as a HEDM candidate.

In short, we constructed the first global potential energy surface of singlet C_3_O_3_ through the thorough isomeric and transition state search strategies. The detailed isomerization/fragmentation and stability data presented in this work should provide an important base for future laboratory study of C_3_O_3_ isomers.

## Conclusions

The key contribution of our work can be summarized as follow:

1. We built up an extensive singlet potential energy surface of the ever elusive molecule, C_3_O_3_, covering 22 isomers and 46 transition states, among which seven isomers have the destruction barriers of 10–25 kcal/mol.
2. We for the first time identified the global isomer of C_3_O_3_, i.e., a three-membered ring structure **01** that has the destruction barrier of 10.3 kcal/mol against decomposition to three CO. Careful examination of its electronic structure indicates that **01** could be partly viewed as a CO-stabilized cyclic carbene.
3. With good kinetic stability, rich oxygen density and the active carbene center, the four-membered ring isomers **02** and **04** can functionalize nano-materials in alkali metal-ion batteries. In particular, with comparable destruction barrier to known HEDM molecules like N_2_CO and ${\text{N}}_{5}^{-}$, **04** can act as a suitable HEDM candidate.

## Data Availability

All datasets generated for this study are included in the manuscript and/or the Supplementary Files.

## Author Contributions

JX formulated the problem, did *ab initio* calculations, searched for literatures, wrote, and finalized the manuscript. XH did composite CBS-QB3 and W1BD calculations, participated in data collection, and analysis. FH formulated the problem, did *ab initio* calculations, and wrote the first manuscript draft. YD formulated the problem, generated key concepts, guided the whole research, and finalized the manuscript.

### Conflict of Interest Statement

The authors declare that the research was conducted in the absence of any commercial or financial relationships that could be construed as a potential conflict of interest.
