# Supplementary material for: Global Isomeric Survey of Elusive Cyclopropanetrione: Unknown but Viable Isomers
Source: Front Chem. 2019 Apr 3;7:193. doi: 10.3389/fchem.2019.00193 (PMC6456661; doi:10.3389/fchem.2019.00193)
Supplement: Supplementary file 1 [file Table_1.docx]

Supplementary Material

[**SI_1**: Flow chart of our strategies for constructing C_3_O_3_ PES………………………..2](#_Toc444452958)

[**SI_2**: Cartesian coordinates and other informations for singlet C_3_O_3_ isomers …..…...3](#_Toc444452959)

[**SI_3**: Cartesian coordinates and other informations for singlet C_3_O_3_ transition states 16](#_Toc444452960)

**SI_4**: Energies for singlet C_3_O_3_ isomers……………………………………………..41

**SI_5**: Energies for singlet C_3_O_3_ transition states………………………………....….42

**SI_6**: Energies for singlet C_3_O_3_ products………………………………………….....44

[**SI_7**: Diagnostic values for key species and associated key transition states…….….44](#_Toc444452965)

[**SI_8**: The natural molecular orbitals for **01**, **02**, **04**……………………….…………46](#_Toc444452966)

[**SI_9**:](#_Toc444452968) Possible intersystem crossing pathways of singlet and triplet isomers **21**….....47

[**SI_10**:](#_Toc444452968) Geometric parameter at the B3LYP, B3LYP-D3BJ and B2PLYP-D3 methods………………………………………………………………………48

[**SI_11**:](#_Toc444452968) RE values for singlet C_3_O_3_ isomers (**01**, **02**, **04** ) with respect to 3CO as well as the destruction barriers at the CCSD(T)//B3LYP+GFEC, CCSD(T)//B3LYP-D3BJ+GFEC and CCSD(T)//B2PLYP-D3+GFEC levels………………………………………………………………………….50

SI_1: Flow chart of our strategies for constructing C_3_O_3_ PES





SI_2: The Cartesian coordinates, total energy (a.u. HF), total energy with zero-point energies correction (a.u. HF+ZPE) and lowest frequencies (lf) of the of the singlet C_3_O_3_ isomers at the B3LYP/aug-cc-VTZ level on the potential energy surface.

01b mol=C3O3

RB3LYP/Aug-CC-pVTZ HF=-339.998983898 HF+ZPE=-339.976504 lf=121.7976

6 1.167746 -0.318554 0.000000

6 0.000000 0.432168 0.000000

6 -1.167163 -0.319257 0.000000

8 -0.000962 1.713411 0.000000

8 2.218669 -0.779235 0.000000

8 -2.218144 -0.779945 0.000000

01 mol=C3O3

RB3LYP/Aug-CC-pVTZ HF=-339.998505836 HF+ZPE=-339.974373 lf=83.6167

8 -0.780449 1.174206 -0.070857

8 -2.165124 -0.768558 -0.062856

8 2.597671 -0.291235 -0.078695

6 -1.184650 -0.125401 0.027659

6 1.457066 -0.103877 0.000960

6 0.191453 0.076726 0.254592

02 mol=C3O3

RB3LYP/Aug-CC-pVTZ HF=-339.931158142 HF+ZPE=-339.907160 lf=114.1912

6 -0.000168 0.020000 0.000000

8 0.000007 0.980592 0.996708

6 -0.000079 -1.293568 0.000000

8 0.000007 -2.454182 0.000000

8 0.000007 0.980592 -0.996708

6 0.000219 1.930898 0.000000

03 mol=C3O3

RB3LYP/Aug-CC-pVTZ HF=-339.915471997 HF+ZPE=-339.892451 lf=211.1569

8 -2.054495 -0.580556 0.032652

6 -0.989095 -0.084737 0.061768

6 0.425591 -0.004688 0.203417

8 1.527042 -0.400597 -0.594849

8 -0.411904 1.142758 -0.133119

6 1.815980 -0.126049 0.661903

04 mol=C3O3

RB3LYP/Aug-CC-pVTZ HF=-339.911423759 HF+ZPE=-339.887105 lf=170.7780

6 -0.000009 -2.356650 0.000000

6 0.000005 -1.023441 0.000000

8 1.017557 -0.079164 0.000000

6 0.000000 0.872093 0.000000

8 -0.000001 2.039329 0.000000

8 -1.017553 -0.079166 0.000000

05mol=C3O3

RB3LYP/Aug-CC-pVTZ HF=-339.921325699 HF+ZPE=-339.897444 lf=92.4658

8 2.299533 1.518008 0.000000

8 -0.710306 -1.726608 0.000000

8 -1.977755 -0.809523 0.000000

6 -0.718594 -0.392403 0.000000

6 1.236632 1.057707 0.000000

6 0.000000 0.692192 0.000000

06 mol=C3O3

RB3LYP/Aug-CC-pVTZ HF=-339.887730628 HF+ZPE=-339.865580 lf=198.1927

8 2.055610 0.000001 -0.000062

6 0.898179 -0.000012 -0.000030

8 -1.025020 -1.039632 0.000050

6 -0.452792 0.000017 0.794334

8 -1.025007 1.039639 0.000006

6 -0.452832 -0.000016 -0.794297

07 mol=C3O3

RB3LYP/Aug-CC-pVTZ HF=-339.887306212 HF+ZPE=-339.865250 lf=183.4254

8 1.887353 -0.000065 -0.000067

6 0.725444 0.000018 0.000059

8 -0.153238 -1.137113 0.000020

8 -0.153089 1.137092 -0.000015

6 -1.416773 -0.832064 -0.000334

6 -1.416705 0.832160 0.000358

08 mol=C3O3

RB3LYP/Aug-CC-pVTZ HF=-339.867058154 HF+ZPE=-339.843949 lf=266.3641

8 0.981539 1.165837 0.000000

6 -0.067951 0.254015 0.000000

8 -0.220043 -0.713372 0.997196

6 -0.433946 -1.628496 0.000000

8 -0.220043 -0.713372 -0.997196

6 -0.220043 1.722358 0.000000

09 mol=C3O3

RB3LYP/Aug-CC-pVTZ HF=-339.827083411 HF+ZPE=-339.804153 lf=196.4323

8 -1.292308 0.211805 0.000000

8 -0.766949 -1.197571 0.000000

8 0.342225 1.832501 0.000000

6 0.000000 0.709431 0.000000

6 1.769010 -1.128292 0.000000

6 0.520366 -0.710119 0.000000

10 mol=C3O3

RB3LYP/Aug-CC-pVTZ HF=-339.789542262 HF+ZPE=-339.768050 lf=169.0283

8 0.433869 -1.887630 0.000000

8 0.983856 1.522366 0.000000

6 0.000000 0.615514 0.000000

6 -0.889274 -1.942553 0.000000

6 -0.291651 -0.645500 0.000000

8 -0.532031 1.844669 0.000000

11 mol=C3O3

RB3LYP/Aug-CC-pVTZ HF=-339.758402637 HF+ZPE=-339.736902 lf=175.8099

8 -0.436762 1.195682 -0.000067

8 1.348811 -0.304026 -0.776047

8 1.348818 -0.303944 0.776065

6 0.313853 0.048948 -0.000003

6 -2.206116 -0.774988 0.000064

6 -1.122226 -0.057576 0.000003

12 mol=C3O3

RB3LYP/Aug-CC-pVTZ HF=-339.736983711 HF+ZPE=-339.715746 lf=367.5008

6 -1.038202 -0.738993 -0.500575

8 -1.540191 0.373548 0.016119

6 -0.197654 0.437089 0.485413

8 1.430803 -0.440131 -0.130409

6 0.221028 -0.902018 0.442998

8 0.870509 0.969525 -0.206587

13 mol=C3O3

RB3LYP/Aug-CC-pVTZ HF=-339.747086884 HF+ZPE=-339.724376 lf=119.4370

6 0.000000 0.159621 0.000000

8 -1.002460 -0.739556 0.000000

8 1.003175 -0.738758 0.000000

8 0.000780 -1.806501 0.000000

6 -0.001462 2.741899 0.000000

6 -0.000531 1.478233 0.000000

14 mol=C3O3

RB3LYP/Aug-CC-pVTZ HF=-339.715243240 HF+ZPE=-339.694622 lf=208.4328

8 1.200736 -0.001111 0.863482

6 0.275310 0.000265 -0.167828

6 -0.864958 -1.020604 -0.240734

8 -1.663201 -0.000261 0.225657

8 1.553343 0.000737 -0.603736

6 -0.864855 1.021187 -0.238643

15 mol=C3O3

RB3LYP/Aug-CC-pVTZ HF=-339.737195732 HF+ZPE=-339.717128 lf=137.6305

8 2.493832 0.264441 0.467846

8 -2.019756 0.642303 -0.149613

6 0.379018 -0.181151 -0.201276

8 1.565340 -0.208593 -0.475114

6 -0.858529 -0.235090 0.020747

6 -2.239711 -0.514628 0.389705

16 mol=C3O3

RB3LYP/Aug-CC-pVTZ HF=-339.691849185 HF+ZPE=-339.671645 lf=114.1366

8 -2.020598 -0.000001 -0.000108

6 -0.834927 -0.000001 0.000036

6 0.230379 1.130582 -0.001786

6 0.230381 -1.130581 0.001862

8 1.150576 -0.002074 -0.716589

8 1.150647 0.002074 0.716612

u17mol=C3O3

UB3LYP/Aug-CC-pVTZ HF=-339.680783324 HF+ZPE=-339.660924 lf=257.0270

6 0.882667 0.022901 1.222476

6 0.368585 -0.798578 0.067224

8 -1.073900 0.741743 -0.036137

8 -1.073948 -0.742448 -0.009174

8 0.933173 -0.017218 -0.950593

6 0.368316 0.799574 0.038173

18 mol=C3O3

RB3LYP/Aug-CC-pVTZ HF=-339.701321876 HF+ZPE=-339.681331 lf=133.4238

8 0.067986 -1.182812 0.000000

8 -2.184967 0.597335 0.000000

6 0.000000 0.137556 0.000000

8 -0.823861 1.041040 0.000000

6 2.616040 -0.401293 0.000000

6 1.305082 -0.343680 0.000000

19 mol=C3O3

RB3LYP/Aug-CC-pVTZ HF=-339.700174778 HF+ZPE=-339.680245 lf=131.4883

6 0.000000 0.375057 0.000000

8 1.190678 0.659776 0.000000

6 -1.085383 -0.484607 0.000000

6 -1.796343 -1.586199 0.000000

8 2.129653 -0.411001 0.000000

8 -1.159036 1.023036 0.000000

u20 mol=C3O3

UB3LYP/Aug-CC-pVTZ HF=-339.675321706 HF+ZPE=-339.656227 lf=118.4078

6 1.849572 0.028754 0.127614

8 0.637105 0.479350 0.019905

6 -0.379492 -0.436386 0.087387

8 -1.435666 0.379834 -0.059462

8 -2.585230 -0.312652 -0.021317

6 3.041641 -0.321077 -0.133835

u21 mol=C3O3

UB3LYP/Aug-CC-pVTZ HF=-339.669649548 HF+ZPE=-339.651164 lf=117.8791

6 -2.317460 -1.036037 -0.036202

6 -1.563552 -0.032090 0.123805

8 -0.746875 0.976270 -0.048564

8 0.940106 -0.351106 -0.088890

6 0.604456 0.945916 0.026435

8 2.264186 -0.533506 0.051926

SI_3: The Cartesian coordinates, total energy (a.u. HF), total energy with zero-point energies correction (a.u. HF+ZPE) and imaginary frequencies (if) of the of the singlet C_3_O_3_ transition states at the B3LYP/aug-cc-VTZ level on the potential energy surface.

ts01b/P1 mol=C3O3

RB3LYP/Aug-CC-pVTZ HF=-339.998592276 HF+ZPE=-339.976943 lf=-107.2922

6 0.000000 0.494372 -0.069428

8 -0.000008 1.742591 -0.014301

6 -1.153851 -0.324543 0.142738

6 1.153858 -0.324534 0.142738

8 -2.171564 -0.813284 -0.073867

8 2.171568 -0.813278 -0.073868

ts01/P1 mol=C3O3

RB3LYP/Aug-CC-pVTZ HF=-339.985193204 HF+ZPE=-339.962235 lf=-388.5775

8 -0.667716 1.422109 -0.000102

8 -2.105021 -0.882697 -0.000067

8 2.484232 -0.440761 -0.000057

6 -1.101313 -0.317025 0.000067

6 1.380346 -0.094275 0.000036

6 0.105640 0.279766 0.000198

ts01/P2 mol=C3O3

RB3LYP/Aug-CC-pVTZ HF=-339.950172609 HF+ZPE=-339.928460 lf=-148.6845

8 -0.607832 0.914191 0.268939

8 -2.417603 -0.416897 -0.347450

8 2.609412 0.101766 -0.374179

6 -1.484429 0.175733 -0.036773

6 1.568696 -0.216462 0.040047

6 0.470430 -0.758019 0.600312

ts01/P3 mol=C3O3

RB3LYP/Aug-CC-pVTZ HF=-339.945643562 HF+ZPE=-339.925617 lf=-189.9483

6 -1.225330 -0.169842 0.134402

6 1.673345 -0.275721 -0.265407

6 -0.111960 0.608842 0.620961

8 2.762762 -0.250168 0.023247

8 -2.036152 -0.999721 -0.058766

8 -0.978651 1.127429 -0.331948

ts01/05 mol=C3O3

RB3LYP/Aug-CC-pVTZ HF=-339.916143606 HF+ZPE=-339.893933 lf=-698.2910

6 0.873454 -0.076451 0.000009

6 -1.622249 -0.078655 0.000005

6 -0.392599 -0.440820 0.000031

8 1.597061 0.997632 0.000018

8 -2.762662 0.136989 -0.000028

8 2.021647 -0.687677 -0.000023

ts03/P3 mol=C3O3

RB3LYP/Aug-CC-pVTZ HF=-339.903370925 HF+ZPE=-339.882596 lf=-416.1606

6 1.926032 0.040761 -0.406368

6 0.337374 0.305619 -0.482501

6 -0.979971 -0.065448 -0.121976

8 -0.432627 1.136239 0.350872

8 1.456662 -0.687888 0.458957

8 -1.986612 -0.659049 -0.051695

ts03/P1 mol=C3O3

RB3LYP/Aug-CC-pVTZ HF=-339.901822134 HF+ZPE=-339.880814 lf=-473.2525

8 -2.062705 -0.578433 0.004506

6 -0.974046 -0.227737 0.082548

6 0.342483 0.182070 0.201663

8 1.471028 -0.522493 -0.578998

8 -0.212998 1.341637 -0.132280

6 1.704464 -0.275281 0.658151

ts03/P4 mol=C3O3

RB3LYP/Aug-CC-pVTZ HF=-339.891695746 HF+ZPE=-339.871204 lf=-408.7535

8 2.014492 -0.683147 -0.065789

6 1.124035 0.073600 -0.012896

6 -0.603321 -0.388741 -0.318573

8 -1.616529 -0.421036 0.575792

8 0.649198 1.179734 0.167158

6 -1.916928 0.214407 -0.571412

ts07/P1 mol=C3O3

RB3LYP/Aug-CC-pVTZ HF=-339.885813914 HF+ZPE=-339.865950 lf=-501.9993

8 -1.935637 0.000000 -0.000271

6 -0.785294 0.000000 -0.000113

8 0.198694 1.187023 0.000109

8 0.198695 -1.187023 -0.000054

6 1.418144 0.863473 0.000260

6 1.418145 -0.863472 0.000141

ts02/P5 mol=C3O3

RB3LYP/Aug-CC-pVTZ HF=-339.884412141 HF+ZPE=-339.865733 lf=-148.1310

6 0.332881 -0.000001 0.597432

8 1.096814 -0.990356 -0.007971

6 -1.658163 -0.000001 -0.352169

8 -2.710975 0.000000 0.048897

8 1.096812 0.990356 -0.007971

6 2.015080 0.000001 -0.289203

1-ts02/P1 mol=C3O3

RB3LYP/Aug-CC-pVTZ HF=-339.882360243 HF+ZPE=-339.860707 lf=-418.5097

6 -0.150878 -0.442847 0.615380

6 -1.291861 -0.083416 0.033349

6 1.648075 0.076280 -0.397344

8 -2.374874 0.248046 -0.220123

8 1.238932 1.074089 0.152962

8 0.981940 -0.984647 -0.121377

2-ts02/P1 mol=C3O3

RB3LYP/Aug-CC-pVTZ HF=-339.881939006 HF+ZPE=-339.862734 lf=-904.7958

6 -0.044994 0.320482 0.187856

8 -0.892048 1.234564 -0.021482

6 1.244395 0.009359 -0.152991

8 2.382837 -0.174261 0.017781

8 -0.908723 -0.998071 0.040542

6 -1.975489 -0.412817 -0.083987

ts06/P1 mol=C3O3

RB3LYP/Aug-CC-pVTZ HF=-339.881175066 HF+ZPE=-339.860488 lf=-487.5353

6 0.894186 -0.009483 -0.008277

6 -0.449314 0.316314 -0.695241

6 -0.421924 -0.416554 0.791427

8 2.051756 0.019862 -0.015362

8 -1.036825 -1.029325 -0.220475

8 -1.032142 1.091754 0.169906

ts02/06 mol=C3O3

RB3LYP/Aug-CC-pVTZ HF=-339.881068225 HF+ZPE=-339.860147 lf=-175.7049

6 1.042938 -0.000001 0.030744

6 -0.233233 -0.000003 -0.678776

6 -0.938453 0.000003 0.835052

8 2.188064 -0.000001 0.092237

8 -1.045749 1.027212 -0.116253

8 -1.045754 -1.027210 -0.116250

ts04/P2 mol=C3O3

RB3LYP/Aug-CC-pVTZ HF=-339.873197220 HF+ZPE=-339.851996 lf=-747.2489

6 -0.840535 -2.154791 0.000000

6 -0.010744 -1.093538 0.000000

8 -0.994058 0.233118 0.000000

6 0.000000 0.990233 0.000000

8 0.515190 2.012517 0.000000

8 1.117328 -0.552064 0.000000

ts04/07 mol=C3O3

RB3LYP/Aug-CC-pVTZ HF=-339.860421611 HF+ZPE=-339.839555 lf=-396.3898

6 1.797775 -0.514684 0.169595

6 -0.731103 -0.103250 0.018137

6 1.165109 0.598447 -0.108706

8 0.026611 1.203337 0.003142

8 -1.897988 -0.151373 0.053204

8 0.197541 -1.037349 -0.115616

ts01/03 mol=C3O3

RB3LYP/Aug-CC-pVTZ HF=-339.844436491 HF+ZPE=-339.823970 lf=-567.2268

6 0.955311 -0.055980 -0.005098

6 -1.278687 -0.977250 -0.710418

6 -0.694354 0.097830 -0.135481

8 -1.612079 0.162228 0.758027

8 0.395689 1.043123 -0.448815

8 1.979688 -0.503802 0.329036

ts08/P5 mol=C3O3

RB3LYP/Aug-CC-pVTZ HF=-339.843293215 HF+ZPE=-339.823401 lf=-515.6776

6 0.049863 0.000070 -0.476922

6 1.746848 0.000027 -0.537328

6 -1.713978 -0.000054 0.222899

8 1.504705 -0.000077 0.643896

8 -0.783400 -0.993606 -0.025239

8 -0.783356 0.993651 -0.025143

ts08/P4mol=C3O3

RB3LYP/Aug-CC-pVTZ HF=-339.816300387 HF+ZPE=-339.795913 lf=-321.9291

8 1.682602 0.065398 -0.547452

6 0.437898 -0.192077 -0.040601

8 -1.530517 -0.855063 -0.139990

6 -1.625468 0.322088 0.119394

8 -0.516028 0.968928 0.084674

6 1.672828 -0.369029 0.724897

ts09/P2 mol=C3O3

RB3LYP/Aug-CC-pVTZ HF=-339.802045223 HF+ZPE=-339.782796 lf=-798.3269

6 -0.697989 -0.038919 -0.018513

6 0.917109 0.379209 0.010789

6 0.921615 1.679827 0.012275

8 -1.679894 0.609408 -0.007271

8 -0.511963 -1.309546 0.023247

8 1.336305 -0.814949 -0.019389

ts01/09 mol=C3O3

RB3LYP/Aug-CC-pVTZ HF=-339.785721590 HF+ZPE=-339.765100 lf=-468.0317

6 0.805864 -0.113026 -0.038121

6 -0.434650 -0.850421 -0.270359

6 -1.614773 -0.886017 0.298897

8 0.218548 1.107506 0.124468

8 -1.245467 0.646442 -0.155852

8 1.959588 -0.366849 0.038571

ts10/P4 mol=C3O3

RB3LYP/Aug-CC-pVTZ HF=-339.776790731 HF+ZPE=-339.757733 lf=-1091.3663

6 -0.643235 -0.223393 -0.001205

6 0.671142 -0.050065 0.008972

6 -1.966993 -0.698879 0.098127

8 1.724322 -0.822127 -0.057148

8 -1.796869 0.621205 -0.081900

8 1.526862 0.930174 0.059627

ts05/10 mol=C3O3

RB3LYP/Aug-CC-pVTZ HF=-339.773737915 HF+ZPE=-339.754617 lf=-227.6011

6 -0.541155 -0.873686 -0.115096

6 0.594301 -0.205988 -0.021384

6 -2.064690 -0.390056 0.233638

8 -1.680136 0.667788 -0.143946

8 1.902803 -0.492197 -0.014014

8 1.285991 0.926707 0.085091

ts12/P1 mol=C3O3

RB3LYP/Aug-CC-pVTZ HF=-339.735081905 HF+ZPE=-339.715561 lf=-556.9242

6 -1.064154 -0.748235 -0.469618

8 -1.547367 0.343066 0.017217

6 -0.154621 0.442105 0.493263

8 1.427530 -0.497717 -0.142574

6 0.271182 -0.897030 0.421499

8 0.830531 1.057021 -0.208500

ts05/11 mol=C3O3

RB3LYP/Aug-CC-pVTZ HF=-339.734146272 HF+ZPE=-339.714135 lf=-593.6826

8 -0.847597 1.217394 0.000155

8 1.271353 -0.069961 -0.785939

8 1.271328 -0.070181 0.785959

6 0.212359 -0.091638 -0.000010

6 -1.213300 -1.316203 -0.000199

6 -1.259173 -0.028496 -0.000025

ts05/15 mol=C3O3

RB3LYP/Aug-CC-pVTZ HF=-339.732934830 HF+ZPE=-339.714116 lf=-353.9980

6 -0.384884 -0.242035 0.170121

6 0.794203 -0.504779 -0.136277

6 2.288459 -0.379217 -0.332809

8 -1.578553 -0.198552 0.476319

8 -2.469860 0.343511 -0.436914

8 2.025079 0.699564 0.184819

ts11/P2 mol=C3O3

RB3LYP/Aug-CC-pVTZ HF=-339.732278740 HF+ZPE=-339.713946 lf=-726.4870

6 0.409789 0.040751 0.000000

6 -1.125819 0.100337 0.000003

6 -1.909578 -0.934103 0.000012

8 1.186854 -0.322073 -0.958567

8 1.186862 -0.322042 0.958572

8 -0.404510 1.238876 -0.000016

ts09/12 mol=C3O3

RB3LYP/Aug-CC-pVTZ HF=-339.729102723 HF+ZPE=-339.707575 lf=-450.9001

6 -0.292581 -0.444155 -0.320311

6 -0.929682 0.993298 0.475746

6 0.178818 0.850848 -0.432086

8 -1.550110 -0.473696 -0.050428

8 0.862197 -0.976782 0.226948

8 1.470498 0.400485 0.030967

ts05/09 mol=C3O3

RB3LYP/Aug-CC-pVTZ HF=-339.716071101 HF+ZPE=-339.697755 lf=-330.2627

6 -0.292753 0.253043 -0.131890

6 -0.085596 1.639133 -0.177213

6 1.046704 -0.179962 -0.033815

8 -1.447088 -0.684457 -0.587468

8 2.163193 -0.419595 0.048617

8 -1.217371 -0.180108 0.796039

ts14/P7 mol=C3O3

RB3LYP/Aug-CC-pVTZ HF=-339.714479405 HF+ZPE=-339.694533 lf=-281.6260

8 -1.188354 -0.574215 -0.661592

6 -0.290957 0.146887 0.109001

6 0.918976 -0.719703 0.699406

8 1.670883 -0.135202 -0.206988

8 -1.570680 0.361649 0.465602

6 0.822849 1.036507 -0.271103

ts13/P6 mol=C3O3

RB3LYP/Aug-CC-pVTZ HF=-339.710636910 HF+ZPE=-339.691416 lf=-830.8269

6 -0.093630 0.125318 0.038040

6 -2.698104 -0.287284 -0.050191

6 -1.451684 -0.038284 0.083100

8 0.795225 -0.893994 -0.037659

8 1.977093 -0.211677 0.017812

8 0.410245 1.255858 -0.033364

ts15/P1 mol=C3O3

RB3LYP/Aug-CC-pVTZ HF=-339.705132775 HF+ZPE=-339.685522 lf=-340.9882

6 -1.760556 0.339677 0.720109

6 0.528040 -0.996464 0.018820

6 -0.545984 -0.121080 0.065706

8 -1.747467 -0.000849 -0.572669

8 1.617763 -0.408695 0.095116

8 1.463579 0.992945 -0.125923

ts18/13 mol=C3O3

RB3LYP/Aug-CC-pVTZ HF=-339.697719813 HF+ZPE=-339.678444 lf=-364.1836

6 0.028378 -0.185523 0.000043

8 1.098966 -0.789374 -0.000089

8 -0.268455 1.064533 -0.000029

8 2.152934 0.214870 -0.000020

6 -2.655753 -0.225489 -0.000280

6 -1.350553 -0.242360 0.000421

ts09/19 mol=C3O3

RB3LYP/Aug-CC-pVTZ HF=-339.697589594 HF+ZPE=-339.678427 lf=-361.1160

6 0.049463 0.444247 -0.000001

6 -1.043035 -0.391931 0.000000

6 -2.033596 -1.242461 0.000001

8 1.864891 -0.864646 0.000000

8 -0.889124 1.319101 0.000000

8 1.294610 0.438154 -0.000001

ts16/P1 mol=C3O3

RB3LYP/Aug-CC-pVTZ HF=-339.691651469 HF+ZPE=-339.671954 lf=-183.8699

6 -0.226972 -1.117946 -0.219155

6 0.834091 0.000000 0.000000

6 -0.226972 1.117946 0.219155

8 2.019689 0.000000 0.000000

8 -1.152399 0.275382 -0.667262

8 -1.152399 -0.275383 0.667262

ts11/19 mol=C3O3

RB3LYP/Aug-CC-pVTZ HF=-339.690095484 HF+ZPE=-339.670726 lf=-297.4228

6 -0.166383 0.364933 0.078146

6 1.107917 -0.215865 0.041673

6 2.062695 -1.108014 0.117276

8 -1.363277 0.438333 0.324626

8 0.789321 1.218638 -0.246083

8 -1.679215 -0.937761 -0.256364

ts11/18 mol=C3O3

RB3LYP/Aug-CC-pVTZ HF=-339.686574172 HF+ZPE=-339.667474 lf=-412.8892

6 0.120400 -0.113155 -0.123564

6 -2.490758 -0.483776 0.270363

6 -1.276984 -0.053918 0.059182

8 -0.384376 1.099944 -0.240717

8 1.117217 -0.793819 -0.347481

8 2.002665 0.182013 0.433713

ts01/17 mol=C3O3

RB3LYP/Aug-CC-pVTZ HF=-339.674984097 HF+ZPE=-339.655872 lf=-533.7884

6 -0.198038 0.695785 -0.631017

6 -0.507220 -0.362778 0.562604

6 -0.638045 1.091394 0.758424

8 1.127535 0.249281 -0.606381

8 0.963754 -0.596551 0.616961

8 -1.083812 -0.721031 -0.528088

ts17/P2 mol=C3O3

RB3LYP/Aug-CC-pVTZ HF=-339.674663311 HF+ZPE=-339.655851 lf=-412.6494

6 1.197953 -0.652881 -0.836210

6 0.258311 0.938011 -0.414325

6 0.346649 -0.678023 0.243662

8 -1.148912 0.564656 -0.335420

8 0.829234 0.510877 0.810468

8 -1.032507 -0.780863 0.280106

ts11/12 mol=C3O3

RB3LYP/Aug-CC-pVTZ HF=-339.664368031 HF+ZPE=-339.645971 lf=-481.9465

6 0.096572 -0.244486 0.333027

6 -1.500984 0.417409 -0.415150

6 -0.597556 0.906418 0.776809

8 1.329868 -0.622450 0.227696

8 -1.032416 -0.870502 -0.203596

8 1.204024 0.683445 -0.545115

ts09/11 mol=C3O3

RB3LYP/Aug-CC-pVTZ HF=-339.663489661 HF+ZPE=-339.645384 lf=-614.0258

6 -0.009105 -0.390767 -0.069786

6 1.458321 0.478618 -0.280544

6 0.497563 1.379058 -0.092558

8 -1.221539 -0.389155 -0.596636

8 0.780510 -1.245146 0.310404

8 -1.019055 0.534120 0.618398

ts11/17 mol=C3O3

RB3LYP/Aug-CC-pVTZ HF=-339.656673948 HF+ZPE=-339.638740 lf=-368.1454

6 -0.018392 -0.582943 -0.068209

6 1.023927 0.868473 -0.121037

6 1.063532 -0.416888 -0.956587

8 -1.295252 -0.632487 0.060617

8 -1.176258 0.875010 -0.094906

8 0.919710 -0.144005 0.893663

ts09/18 mol=C3O3

RB3LYP/Aug-CC-pVTZ HF=-339.628515380 HF+ZPE=-339.611038 lf=-147.4356

6 -1.234105 1.494543 0.030357

6 -0.048298 -0.469404 0.720333

6 -1.275654 0.208128 -0.115624

8 0.926435 -0.124184 -0.357339

8 -1.046757 -1.073735 -0.167604

8 2.038865 0.272969 0.048644

ts21/P2 mol=C3O3

RB3LYP/Aug-CC-pVTZ HF=-339.652443334 HF+ZPE=-339.636536 lf=-795.9535

6 -2.283405 -1.111985 0.047896

6 -1.576265 -0.015764 -0.017872

6 0.755675 0.897280 0.120321

8 0.916059 -0.329024 -0.044687

8 2.321998 -0.517740 -0.008759

8 -0.910062 1.019616 -0.059312

**SI_4** Energies for singlet C_3_O_3_ isomers.

| Isomers | CCSD(T)//B3LYP+ZPEC | RE | CCSD(T)//B3LYP+GFEC | RE |
| --- | --- | --- | --- | --- |
| 01 | -339.3618097 | 68.4 | -339.3910767 | 86.0 |
| 01b | -339.3592484 | 70.0 | -339.3893904 | 87.1 |
| 02 | -339.3023545 | 105.7 | -339.3310145 | 123.7 |
| 03 | -339.2916483 | 112.4 | -339.3197223 | 130.8 |
| 04 | -339.2871471 | 115.3 | -339.3150951 | 133.7 |
| 05 | -339.2860711 | 115.9 | -339.3152651 | 133.6 |
| 06 | -339.2746383 | 123.1 | -339.3024573 | 141.6 |
| 07 | -339.2687798 | 126.8 | -339.2970088 | 145.1 |
| 08 | -339.2510252 | 137.9 | -339.2785562 | 156.6 |
| 09 | -339.2011141 | 169.2 | -339.2294171 | 187.5 |
| 10 | -339.1652593 | 191.7 | -339.1939393 | 209.7 |
| 11 | -339.1380023 | 208.8 | -339.1664003 | 227.0 |
| 12 | -339.1217207 | 219.1 | -339.1491187 | 237.9 |
| 13 | -339.1163073 | 222.5 | -339.1452273 | 240.3 |
| 14 | -339.1012844 | 231.9 | -339.1293074 | 250.3 |
| 15 | -339.1011982 | 231.9 | -339.1305302 | 249.5 |
| 16 | -339.0835069 | 243.0 | -339.1120809 | 261.1 |
| 17 | -339.074888 | 248.5 | -339.102535 | 267.1 |
| 18 | -339.0714819 | 250.6 | -339.1007169 | 268.2 |
| 19 | -339.0687465 | 252.3 | -339.0980845 | 269.9 |
| 20 | -339.0437744 | 268.0 | -339.0731594 | 285.5 |
| 21 | -339.0381173 | 271.5 | -339.0678123 | 288.9 |

**SI_5** Energies for singlet C_3_O_3_ transition states.

| Transition states | CCSD(T)//B3LYP+ZPEC | RE | CCSD(T)//B3LYP+GFEC | RE |
| --- | --- | --- | --- | --- |
| ts01b/P1 | -339.3619477 | 68.3 | -339.3913937 | 85.8 |
| ts01/P1 | -339.3458211 | 78.4 | -339.3747221 | 96.3 |
| ts01/P3 | -339.3259477 | 90.9 | -339.3561897 | 107.9 |
| ts01/P2 | -339.3207228 | 94.2 | -339.3510908 | 111.1 |
| ts01/05 | -339.2834304 | 117.6 | -339.3128804 | 135.1 |
| ts03/P3 | -339.2817085 | 118.7 | -339.3100295 | 136.9 |
| ts03/P1 | -339.2762216 | 122.1 | -339.3045986 | 140.3 |
| ts02/P5 | -339.2726094 | 124.4 | -339.3026847 | 141.5 |
| ts03/P4 | -339.2689543 | 126.7 | -339.2979563 | 144.5 |
| ts06/P1 | -339.2669504 | 127.9 | -339.2949824 | 146.3 |
| ts07/P1 | -339.2670775 | 127.9 | -339.2957845 | 145.8 |
| ts02/06 | -339.2643797 | 129.5 | -339.2922587 | 148.0 |
| ts02/P1 | -339.2573621 | 133.9 | -339.2864921 | 151.7 |
| ts02/P1 | -339.2558361 | 134.9 | -339.2854061 | 152.3 |
| ts04/P2 | -339.247741 | 140.0 | -339.27643 | 158.0 |
| ts04/07 | -339.2371747 | 146.6 | -339.2653627 | 164.9 |
| ts08/P5 | -339.2287399 | 151.9 | -339.2568649 | 170.3 |
| ts01/03 | -339.2186829 | 158.2 | -339.2473329 | 176.2 |
| ts08/P4 | -339.2004113 | 169.7 | -339.2290373 | 187.7 |
| ts09/P2 | -339.1795134 | 182.8 | -339.2083794 | 200.7 |
| ts01/09 | -339.1643319 | 192.3 | -339.1924569 | 210.7 |
| ts10/P4 | -339.1573161 | 196.7 | -339.1861291 | 214.6 |
| ts05/10 | -339.1549067 | 198.2 | -339.1841607 | 215.9 |
| ts12/P1 | -339.1207993 | 219.6 | -339.1483153 | 238.4 |
| ts05/11 | -339.118779 | 220.9 | -339.14704 | 239.2 |
| ts09/12 | -339.1137495 | 224.1 | -339.1409865 | 243.0 |
| ts13/P6 | -339.1118747 | 225.2 | -339.1410417 | 242.9 |
| ts11/P2 | -339.1114277 | 225.5 | -339.1402787 | 243.4 |
| ts14/P7 | -339.1009659 | 232.1 | -339.1285789 | 250.8 |
| ts05/15 | -339.0996003 | 232.9 | -339.1291033 | 250.4 |
| ts05/09 | -339.093731 | 236.6 | -339.122849 | 254.3 |
| ts16/P1 | -339.0828397 | 243.5 | -339.1107197 | 262.0 |
| ts15/P1 | -339.0806766 | 244.8 | -339.1091056 | 263.0 |
| ts18/13 | -339.0669742 | 253.4 | -339.0961332 | 271.1 |
| ts01/17 | -339.0664341 | 253.8 | -339.0938071 | 272.6 |
| ts17/P2 | -339.066451 | 253.7 | -339.093937 | 272.5 |
| ts09/19 | -339.0638985 | 255.3 | -339.0932825 | 272.9 |
| ts11/19 | -339.0624171 | 256.3 | -339.0911301 | 274.3 |
| ts11/18 | -339.0593122 | 258.2 | -339.0880232 | 276.2 |
| ts09/11 | -339.0562565 | 260.1 | -339.0842315 | 278.6 |
| ts11/12 | -339.0508199 | 263.6 | -339.0790709 | 281.8 |
| ts11/17 | -339.0448902 | 267.3 | -339.0728892 | 285.7 |
| uts21/P2 | -339.0146759 | 286.2 | -339.0452419 | 303.0 |
| ts09/18 | -339.009204 | 289.7 | -339.037956 | 307.6 |
| ts20/21 | -339.0268748 | 278.6 | -339.0567578 | 295.8 |
| ts19/20 | -339.0189524 | 283.6 | -339.0488154 | 300.8 |

**SI_6** Energies for singlet C_3_O_3_ products.

| Products | CCSD(T)//B3LYP+ZPEC | RE | CCSD(T)//B3LYP+GFEC | RE |
| --- | --- | --- | --- | --- |
| P1:3CO | -339.4708221 | 0.0 | -339.5281791 | 0.0 |
| P2:(u) CCO+CO_2_ | -339.3375326 | 83.6 | -339.3753106 | 95.9 |
| P3:O-c-CCO+CO | -339.3315792 | 87.4 | -339.3759402 | 95.5 |
| P4:c-CCO+CO_2_ | -339.2960441 | 109.7 | -339.3356121 | 120.8 |
| P5:c-OCCO+CO | -339.2778069 | 121.1 | -339.3220089 | 129.4 |
| P6:(u) O_2_+CCCO | -339.1994528 | 170.3 | -339.2364618 | 183.1 |
| P7:Y-OCCO+CO | -339.1618707 | 193.9 | -339.2064857 | 201.9 |

SI_7 : The T1 diagnostic values (T1Diag) for the species and associated key transition states at the CCSD/aug-cc-pVTZ level.

| Isomers | T1Diag | Relevant transition states | T1Diag |
| --- | --- | --- | --- |
| 01 | 0.019 | ts01/P1  ts01/P3  ts01/P4 | 0.022  0.020  0.021 |
| 01b | 0.023 | 01b-ts | 0.022 |
| 02 | 0.022 | ts02/P5  ts02/06 | 0.022  0.023 |
| 04 | 0.018 | ts04/P2  ts04/07 | 0.022  0.027 |
| 08 | 0.021 | ts08/P1 | 0.026 |
| 09 | 0.023 | uts09/P2 | 0.030 |
| 11 | 0.021 | ts11/P2 | 0.029 |
| 20(u) | 0.039 | ts20/21  ts19/20 | 0.028  0.028 |
| 21(u) | 0.038 | ts21/P2 | 0.030 |

SI_8: The natural molecular orbitals for **01**, **02**, **04** at B3LYP/aug-cc-pVTZ.





SI_9**:** Possible intersystem crossing pathways of singlet and triplet isomers **21** at the [CCSD(T)//B3LYP+ZPEC], (B3LYP/aug-cc-pVTZ+ZPEC) levels.

**

**

SI_10**:** Geometric parameter of Bond length (Å), Bond angle (°) and Dihedral angle (°) at the B3LYP/aug-cc-pVTZ, B3LYP-D3BJ/aug-cc-pVTZ and B2PLYP-D3/aug-cc-pVTZ levels.

| Structure | Parameter | B3LYP/aug-cc-pVTZ | B3LYP-D3BJ/aug-cc-pVTZ | B2PLYP-D3/aug-cc-pVTZ |
| --- | --- | --- | --- | --- |
| 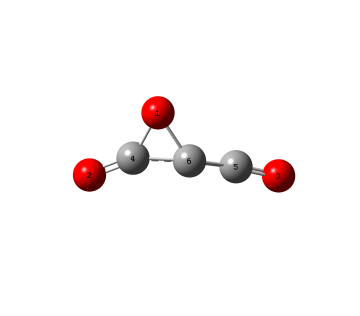  **01** | 1O-4C | 1.365 | 1.365 | 1.363 |
|  | 2O-4C | 1.176 | 1.176 | 1.180 |
|  | 1O-6C | 1.502 | 1.501 | 1.521 |
|  | 6C-5C | 1.303 | 1.303 | 1.315 |
|  | 5C-3O | 1.159 | 1.159 | 1.161 |
|  | 4C-6C | 1.409 | 1.409 | 1.416 |
|  | ∠416 | 58.7 | 58.6 | 58.5 |
|  | ∠241 | 139.7 | 139.7 | 139.6 |
|  | ∠146 | 65.5 | 65.5 | 66.3 |
|  | ∠246 | 155.8 | 154.8 | 154.1 |
|  | ∠164 | 55.8 | 55.8 | 55.2 |
|  | ∠165 | 133.4 | 133.4 | 129.2 |
|  | ∠653 | 172.6 | 172.6 | 170.2 |
|  | ∠2416 | 179.7 | 179.7 | 179.3 |
|  | ∠4165 | 148.2 | 148.1 | 137.7 |
|  | ∠1653 | 128.9 | 129.0 | 130.3 |
|  |  |  |  |  |
| 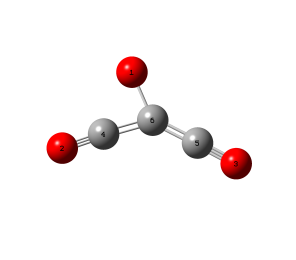  **01-TS** | 1O-4C | 1.792 | 1.785 | 1.794 |
|  | 2O-4C | 1.152 | 1.152 | 1.157 |
|  | 1O-6C | 1.380 | 1.382 | 1.385 |
|  | 6C-5C | 1.328 | 1.327 | 1.330 |
|  | 5C-3O | 1.157 | 1.157 | 1.163 |
|  | 4C-6C | 1.346 | 1.346 | 1.347 |
|  | ∠416 | 48.1 | 48.3 | 48.1 |
|  | ∠246 | 176.9 | 176.5 | 176.4 |
|  | ∠165 | 140.5 | 140.6 | 140.6 |
|  | ∠465 | 137.3 | 137.6 | 137.3 |
|  | ∠653 | 178.9 | 178.8 | 179.0 |
|  | ∠2416 | -180.0 | -180.0 | -180.0 |
|  | ∠4615 | -180.0 | -180.0 | -180.0 |
|  | ∠1653 | 179.9 | 180.0 | 180.0 |

| Structure | Parameter | B3LYP/aug-cc-pVTZ | B3LYP-D3BJ/aug-cc-pVTZ | B2PLYP-D3/aug-cc- pVTZ |
| --- | --- | --- | --- | --- |
| 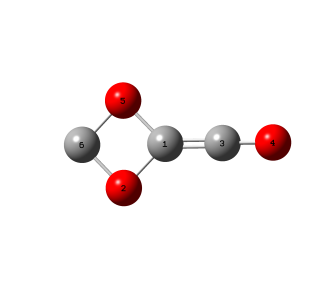  **02** | 6C-5O | 1.377 | 1.377 | 1.379 |
|  | 6C-2O | 1.377 | 1.377 | 1.379 |
|  | 1C-5O | 1.384 | 1.384 | 1.385 |
|  | 1C-2O | 1.384 | 1.384 | 1.385 |
|  | 1C-3C | 1.314 | 1.313 | 1.315 |
|  | 3C-4O | 1.161 | 1.160 | 1.165 |
|  | ∠265 | 92.7 | 92.7 | 92.9 |
|  | ∠651 | 87.6 | 87.6 | 87.3 |
|  | ∠512 | 92.1 | 92.1 | 92.4 |
|  | ∠126 | 87.6 | 87.6 | 87.3 |
|  | ∠134 | 180.0 | 180.0 | 180.0 |
|  | ∠213 | 133.9 | 134.0 | 133.8 |
|  | ∠513 | 133.9 | 134.0 | 133.8 |
|  | ∠6521 | 180.0 | 180.0 | 180.0 |
|  | ∠5621 | 0.0 | 0.0 | 0.0 |
|  | ∠5134 | 90.0 | 90.0 | 90.0 |
|  | ∠2134 | 90.0 | 90.0 | 90.0 |
|  | ∠6513 | -180.0 | -180.0 | -180.0 |
|  | ∠6213 | 180.0 | 180.0 | 180.0 |
|  |  |  |  |  |
| 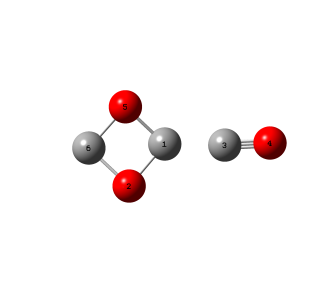  **02-TS** | 6C-5O | 1.380 | 1.380 | 1.380 |
|  | 6C-2O | 1.380 | 1.380 | 1.380 |
|  | 1C-5O | 1.390 | 1.389 | 1.395 |
|  | 1C-2O | 1.390 | 1.389 | 1.395 |
|  | 1C-3C | 2.206 | 2.247 | 2.095 |
|  | 3C-4O | 1.127 | 1.126 | 1.132 |
|  | ∠265 | 91.8 | 91.7 | 92.1 |
|  | ∠651 | 86.7 | 86.7 | 86.7 |
|  | ∠512 | 90.9 | 91.0 | 90.9 |
|  | ∠126 | 86.7 | 86.7 | 86.7 |
|  | ∠134 | 133.6 | 133.4 | 135.2 |
|  | ∠213 | 108.0 | 107.2 | 107.5 |
|  | ∠513 | 108.0 | 107.2 | 107.5 |
|  | ∠6521 | 158.6 | 158.3 | 159.1 |
|  | ∠5621 | -14.8 | -15.0 | -14.6 |
|  | ∠5134 | -131.5 | -131.7 | -131.7 |
|  | ∠2134 | 131.5 | 131.7 | 131.7 |
|  | ∠6513 | -124.0 | -123.3 | -123.1 |
|  | ∠6213 | 124.0 | 123.3 | 123.1 |

| Structure | Parameter | B3LYP/aug-cc-pVTZ | B3LYP-D3BJ/aug-cc-pVTZ | B2PLYP-D3/aug-cc- pVTZ |
| --- | --- | --- | --- | --- |
| 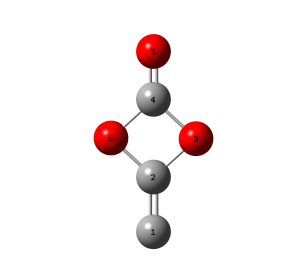  **04** | 5O-4C | 1.167 | 1.167 | 1.169 |
|  | 4C-6O | 1.393 | 1.394 | 1.397 |
|  | 4C-3O | 1.393 | 1.394 | 1.397 |
|  | 6O-2C | 1.388 | 1.388 | 1.387 |
|  | 3O-2C | 1.388 | 1.388 | 1.387 |
|  | 2C-1C | 1.333 | 1.333 | 1.338 |
|  | ∠546 | 133.1 | 133.1 | 133.1 |
|  | ∠543 | 133.1 | 133.1 | 1.3 |
|  | ∠462 | 85.9 | 86.0 | 85.7 |
|  | ∠432 | 85.9 | 86.0 | 85.7 |
|  | ∠643 | 93.9 | 93.8 | 93.8 |
|  | ∠623 | 94.3 | 94.2 | 97.7 |
|  | ∠621 | 132.9 | 132.9 | 132.6 |
|  | ∠321 | 132.9 | 132.9 | 132.6 |
|  | ∠5462 | 180.0 | 180.0 | 180.0 |
|  | ∠5432 | 180.0 | 180.0 | 180.0 |
|  | ∠6432 | 0.0 | 0.0 | 0.0 |
|  | ∠4621 | 180.0 | 180.0 | 180.0 |
|  | ∠4321 | 180.0 | 180.0 | 180.0 |
|  | ∠6321 | 180.0 | 180.0 | 180.0 |
|  |  |  |  |  |
| 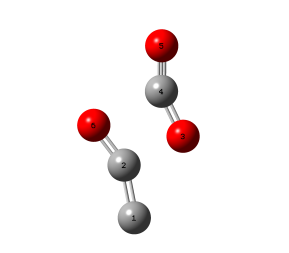  **04-TS** | 5O-4C | 1.145 | 1.145 | 1.149 |
|  | 4C-6O | 1.905 | 1.904 | 1.903 |
|  | 4C-3O | 1.250 | 1.250 | 1.254 |
|  | 6O-2C | 1.251 | 1.252 | 1.254 |
|  | 3O-2C | 1.651 | 1.648 | 1.641 |
|  | 2C-1C | 1.347 | 1.347 | 1.351 |
|  | ∠546 | 117.3 | 117.5 | 117.4 |
|  | ∠543 | 154.0 | 154.0 | 153.9 |
|  | ∠462 | 79.7 | 79.7 | 79.2 |
|  | ∠432 | 90.7 | 90.8 | 90.5 |
|  | ∠643 | 88.6 | 88.6 | 88.7 |
|  | ∠623 | 100.9 | 101.0 | 101.5 |
|  | ∠621 | 153.7 | 153.5 | 153.7 |
|  | ∠321 | 105.4 | 105.5 | 104.8 |
|  | ∠5462 | 180.0 | 180.0 | 180.0 |
|  | ∠5432 | 180.0 | 180.0 | 180.0 |
|  | ∠6432 | 0.0 | 0.0 | 0.0 |
|  | ∠4621 | 180.0 | 180.0 | 180.0 |
|  | ∠4321 | 180.0 | 180.0 | 180.0 |
|  | ∠6321 | 180.0 | 180.0 | 180.0 |

SI_11**:** RE values for singlet C_3_O_3_ isomers (**01**, **02**, **04** ) with respect to 3CO as well as the destruction barriers at the CCSD(T)//B3LYP+GFEC, CCSD(T)//B3LYP-D3BJ+GFEC and CCSD(T)//B2PLYP-D3+GFEC levels.

|  | CCSD(T)//B3LYP+GFEC | | CCSD(T)//B3LYP-D3BJ+GFEC | | CCSD(T)//B2PLYP-D3+GFEC | |
| --- | --- | --- | --- | --- | --- | --- |
|  | RE | the destruction barrier | RE | the destruction barrier | RE | the destruction barrier |
| **01** | 86.0 | 10.3 | 86.0 | 10.2 | 86.1 | 10.5 |
| **02** | 123.7 | 17.8 | 123.7 | 17.2 | 123.9 | 18.8 |
| **04** | 133.7 | 24.3 | 133.7 | 24.3 | 134.1 | 24.2 |
